# Functional and Radiographic Outcomes of Proximal Fibular Osteotomy in Medial Compartment Knee Osteoarthritis: A Prospective Single-Arm Interventional Study With 12-Month Follow-Up

**DOI:** 10.7759/cureus.105886

**Published:** 2026-03-26

**Authors:** Shailesh Dahal, Adarsha Mahaseth, Bishal Adhikari, Ramchandra Sapkota, Hasham I Awan

**Affiliations:** 1 Orthopaedic Surgery, The Second Xiangya Hospital of Central South University, Changsha, CHN; 2 Internal Medicine, Nepalese Army Institute of Health Sciences, Kathmandu, NPL; 3 Orthopaedics and Trauma, Madan Bhandari Academy of Health Sciences, Hetauda, NPL; 4 Urology, The First Xiangya Hospital of Central South University, Changsha, CHN

**Keywords:** joint-preserving surgery, joint space width, knee alignment, knee osteoarthritis, medial compartment osteoarthritis, proximal fibular osteotomy, tibiofemoral angle, varus deformity, visual analog scale, womac score

## Abstract

Background

Medial compartment knee osteoarthritis (OA) is a common degenerative joint disorder and a major cause of chronic pain and functional impairment, particularly among middle-aged and older adults. In low-resource healthcare settings, access to procedures such as knee arthroplasty remains limited. Proximal fibular osteotomy (PFO) has emerged as a minimally invasive, joint-preserving surgical technique that may improve load distribution across the knee and correct varus alignment in selected patients.

Objective

This study aimed to assess the functional and radiological outcomes of PFO in patients with medial compartment knee OA at the 12-month follow-up.

Methods

A hospital-based prospective single-arm interventional study was conducted among 42 patients with Kellgren-Lawrence grade II-III medial compartment knee OA who underwent PFO at a tertiary care center in Nepal. The primary outcome was the change in the Western Ontario and McMaster Universities Osteoarthritis Index (WOMAC) score at 12 months. Pain intensity was assessed using the Visual Analog Scale (VAS). Radiological parameters included medial joint space width and tibiofemoral varus angle. Patients were evaluated at six and 12 months postoperatively. The Wilcoxon signed-rank test was used for non-normally distributed variables (VAS and WOMAC), and paired t-tests were used for normally distributed radiological parameters. Statistical significance was set at p<0.05.

Results

The mean age of participants was 56.90±9.33 years. Significant improvements were observed at 12 months. The mean VAS score decreased from 7.29±1.13 preoperatively to 3.33±1.76 (p<0.001). The mean WOMAC score improved from 75.36±8.68 to 33.60±12.94 (p<0.001). The medial joint space width increased from 2.04±0.39 mm to 3.74±0.46 mm (p<0.001), and the tibiofemoral varus angle improved from 8.44°±2.29° to 4.40°±2.43° (p<0.001). Excellent functional improvement (≥75% reduction in WOMAC score) was achieved in 61.9% of patients. The overall complication rate was 19%, with most complications being minor and transient.

Conclusions

PFO was associated with significant improvements in pain, functional outcomes, and radiological parameters at 12 months in this cohort, with an acceptable complication profile. These findings suggest that PFO may be a useful joint-preserving option in selected patients with medial compartment knee OA, particularly in resource-limited settings. However, results should be interpreted cautiously due to the single-arm study design and lack of a comparator group.

## Introduction

Knee osteoarthritis (OA) is among the most common degenerative joint conditions and represents a major contributor to chronic pain, reduced functional capacity, and disability globally. Epidemiological data from the Framingham Osteoarthritis Study demonstrated that the prevalence of radiographic knee OA increases substantially with age, rising from approximately 27% in individuals younger than 70 years to nearly 44% in those aged 80 years or older, with women exhibiting higher rates of symptomatic disease than men [1]. Additional longitudinal analyses from the Framingham cohort identified advancing age, obesity, prior knee injury, and occupational knee stress as important risk factors for disease development and progression [1]. Population-based data further suggest that up to 20% of older adults have radiographic knee OA, with approximately 7% experiencing symptomatic disease, underscoring its contribution to the global burden of disability [2].

Biomechanical studies have consistently shown that knee alignment plays a critical role in disease progression. Varus malalignment increases medial tibiofemoral load transmission and is associated with nearly fourfold higher odds of medial OA progression compared with neutrally aligned knees [3]. Alignment deviations, particularly varus deformity, are further linked to mechanical axis deviation, bone deformity, and medial joint space narrowing, reinforcing the load-induced pathophysiological model of medial compartment degeneration [4,5]. Therefore, surgical strategies aimed at modifying load distribution and correcting mechanical alignment form the biomechanical basis for joint-preserving interventions such as proximal fibular osteotomy (PFO).

Given that medial compartment involvement accounts for the majority of knee OA cases, largely due to varus alignment and disproportionate medial loading, surgical strategies aimed at correcting biomechanical imbalance are clinically relevant. Although procedures such as high tibial osteotomy (HTO) and total knee arthroplasty (TKA) are well-established and effective, their cost and limited availability can restrict their use in resource-constrained environments. PFO has emerged as a less invasive surgical option based on the concept of lateral column decompression. Yang et al. demonstrated significant reductions in pain and marked improvement in functional scores following PFO, along with the radiographic evidence of load redistribution and alignment correction [6]. A subsequent systematic review by Ashraf et al. reported consistent short-term improvements in pain, function, and radiographic alignment across multiple clinical studies, with predominantly minor complications, although high-quality randomized trials remain limited [7]. Although previous international studies have reported promising results, evidence from Nepal and comparable healthcare settings remains limited. Therefore, the present study was conducted to assess the functional and radiological outcomes of PFO in patients with medial compartment OA of the knee.

## Materials and methods

Study design and setting

A hospital-based prospective single-arm interventional study was undertaken at the Department of Orthopaedics of Nepal Medical College and Teaching Hospital in Kathmandu, Nepal, during an 18-month study period.

Study population

Patients attending the orthopaedic outpatient department with symptomatic medial compartment knee OA who were scheduled to undergo PFO were assessed for eligibility.

Eligibility criteria

Patients aged between 40 and 75 years with a radiological diagnosis of medial compartment OA (Kellgren-Lawrence grades II-III), presence of varus deformity, and failure of conservative management for at least six months and who were willing to provide written informed consent were included in the study.

Patients with tricompartmental or lateral compartment OA, rheumatoid arthritis or other inflammatory arthropathies, a history of previous knee surgery, severe valgus deformity, or neuromuscular disorders affecting the lower limbs were excluded.

Sample size and sampling technique

The required sample size was determined using the formula for estimating a single population mean: $\mathrm{n}=\mathrm{Z}^{2}\times\mathrm{\sigma}^{2}/\mathrm{d}^{2}$. Here, Z is equal to 1.96 for a 95% confidence interval, σ is equal to 15.18 (standard deviation derived from a previously reported postoperative American Knee Society Score (AKSS) of 71.46±15.18) [8], and d is equal to 5 (absolute precision). Based on this calculation, the minimum required sample size was determined to be 36 patients. After incorporating an additional 15% to compensate for possible loss to follow-up, the final sample size was adjusted to 42 patients.

A consecutive sampling technique was employed. All eligible patients undergoing PFO during the study period were enrolled until the required sample size was achieved.

Preoperative assessment

Baseline demographic information, including age, sex, body mass index (BMI), and the affected side of the knee, was documented. Clinical assessment included recording the duration of symptoms along with findings from physical examination.

Pain intensity was evaluated using the Visual Analog Scale (VAS), which ranges from 0 to 10. Functional status was assessed using the Western Ontario and McMaster Universities Osteoarthritis Index (WOMAC) Likert version (score range 0-96, with higher scores indicating worse pain and functional limitation).

Radiographic assessment was performed using standardized weight-bearing standing anteroposterior (AP) knee radiographs. Medial joint space width was measured in millimeters at the narrowest region of the medial tibiofemoral compartment. The tibiofemoral angle was determined to evaluate the extent of varus alignment. The severity of OA was classified according to the Kellgren-Lawrence grading system. Radiological measurements were performed by independent observers (blinded to time points where applicable) to reduce measurement bias.

Surgical procedure

All surgical procedures were carried out under spinal or regional anesthesia by experienced orthopaedic surgeons following a standardized surgical protocol. Patients were placed in a supine position on the operating table, and the affected limb was prepared and draped using sterile technique.

**Figure 1:**
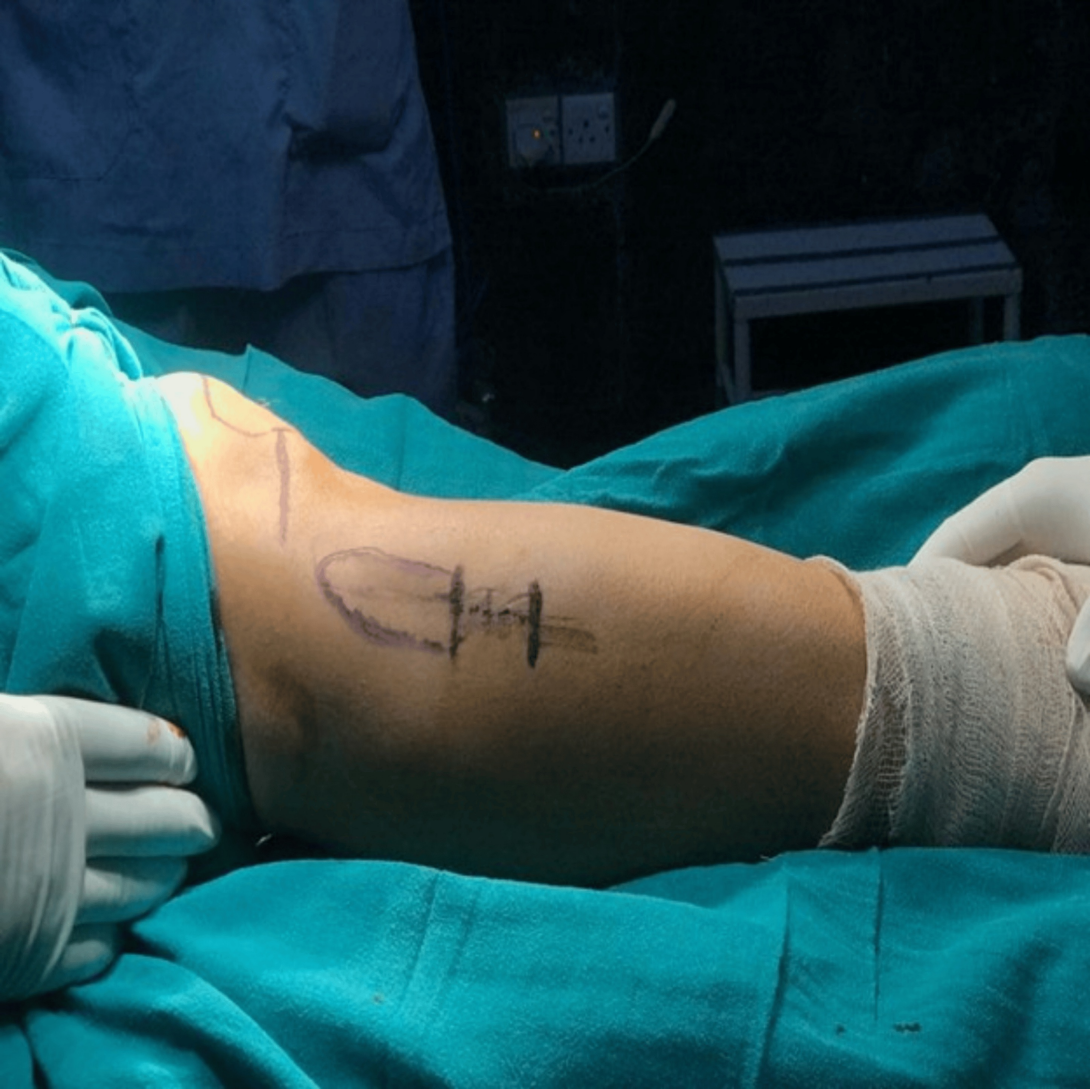
Preoperative surface marking for proximal fibular osteotomy showing the incision site approximately 6-8 cm distal to the fibular head

The surgical site was identified and marked over the lateral aspect of the proximal fibula, approximately 6-8 cm distal to the fibular head (standardized osteotomy level), to minimize the risk of injury to the common peroneal nerve (Figure 1).

**Figure 2:**
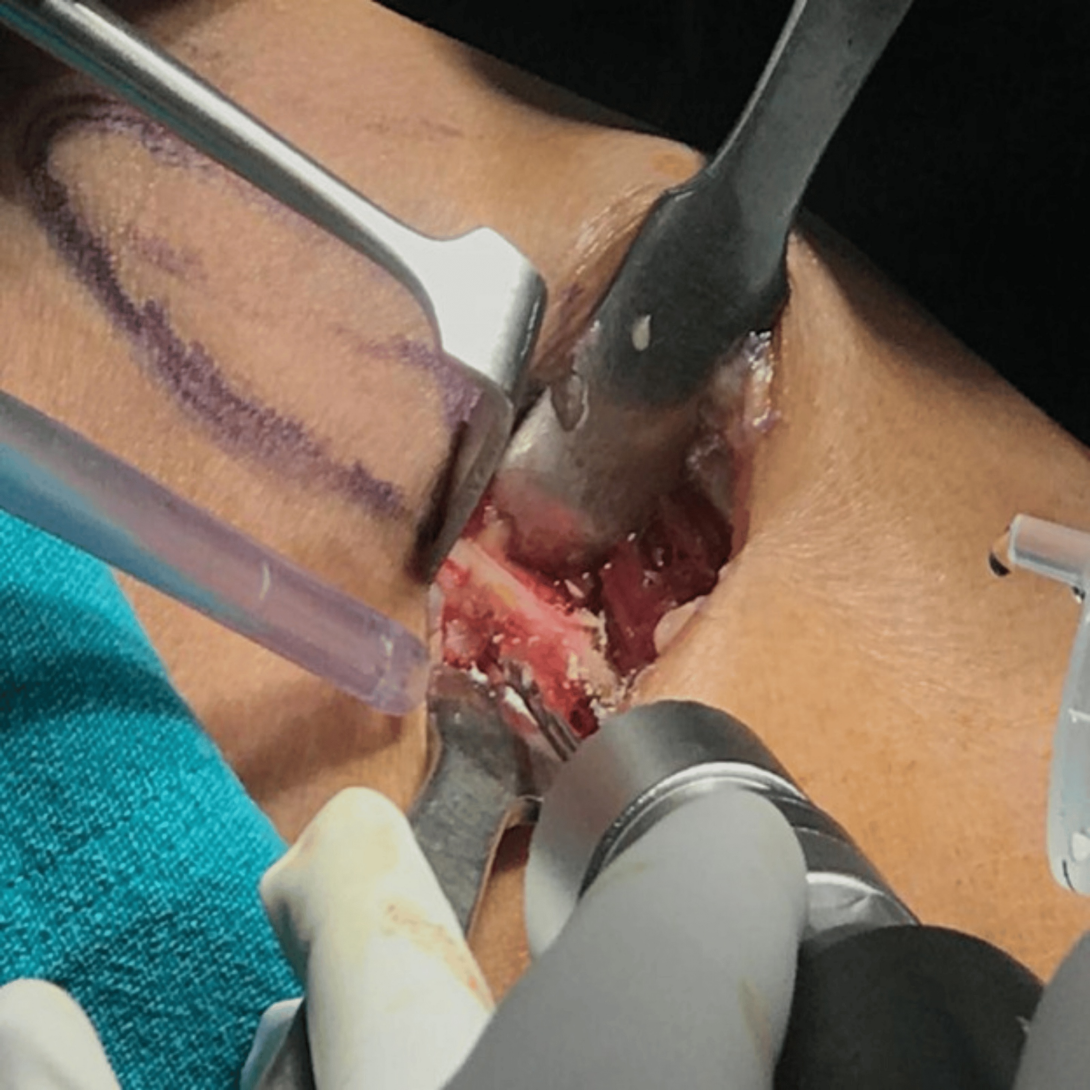
Intraoperative exposure of the proximal fibula following skin incision and soft tissue dissection during proximal fibular osteotomy

A longitudinal skin incision of approximately 2-3 cm was made at the marked location. Blunt and sharp dissection was then carried out through the subcutaneous tissue and fascia to expose the proximal fibula, taking care to avoid injury to the surrounding neurovascular structures (Figure 2).

**Figure 3:**
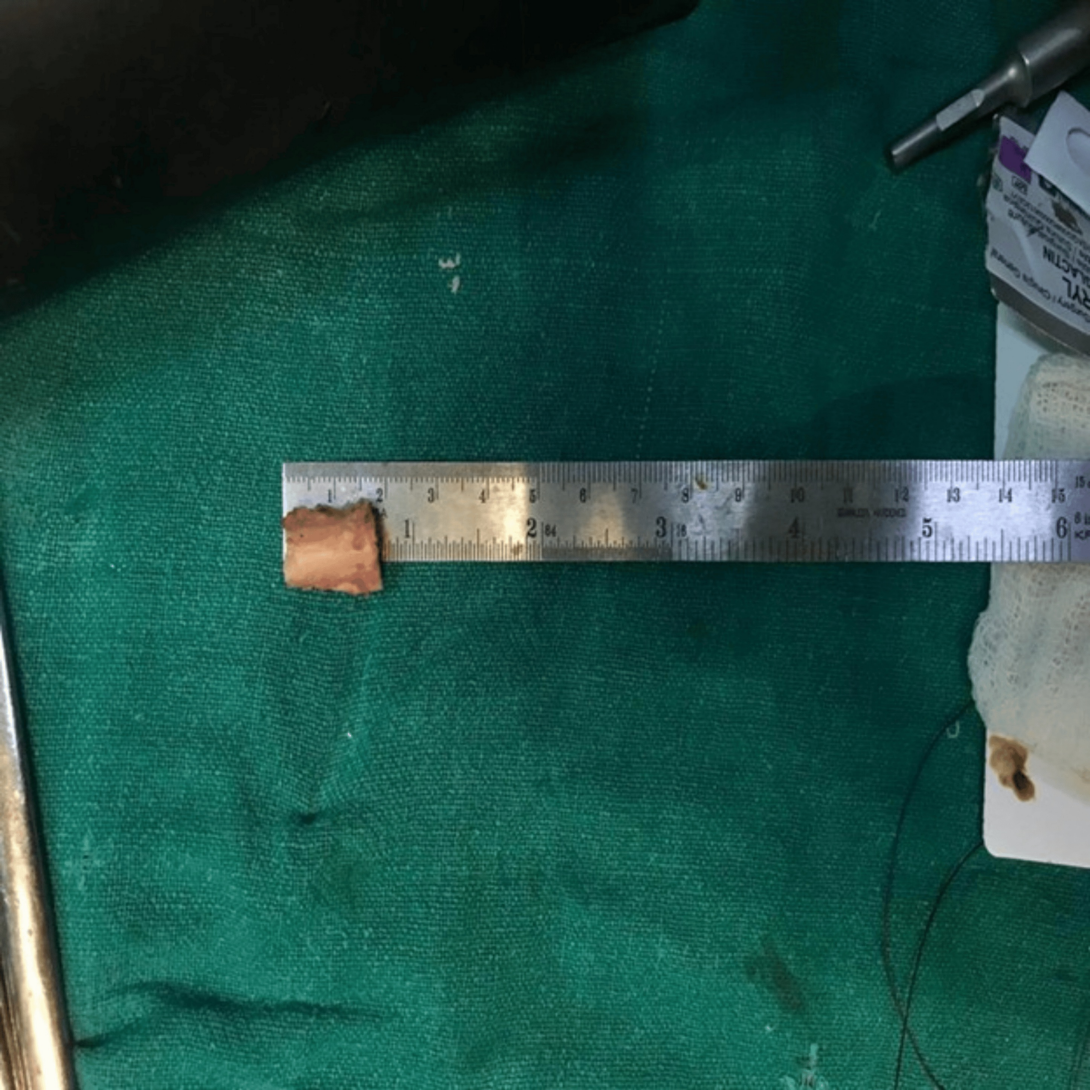
Resected fibular segment measuring approximately 2 cm following proximal fibular osteotomy

After sufficient exposure of the bone, a 1.5-2 cm segment of the fibula was removed using an oscillating saw to perform the PFO (Figure 3). Care was taken to completely excise the bone segment while protecting the surrounding soft tissues.

Hemostasis was achieved, and the wound was irrigated thoroughly before closure in layers. A sterile dressing was applied following wound closure. Early postoperative mobilization was encouraged, and patients were allowed to bear weight as tolerated according to a uniform postoperative rehabilitation protocol.

Follow-up protocol

Patients were followed up at six weeks, three months, six months, and 12 months postoperatively.

At each follow-up visit, pain (VAS) and functional status (WOMAC score) were reassessed. Radiological evaluation was repeated at six and 12 months to measure medial joint space width and tibiofemoral angle. Postoperative complications were documented at each visit. Although assessments were performed at six weeks and three months, outcome analysis primarily focused on the six- and 12-month follow-up periods.

Outcome measures

The primary endpoint of the study was the change in WOMAC total score from baseline to the six-month and 12-month follow-up.

Secondary outcomes included change in VAS pain score, change in medial joint space width, change in tibiofemoral varus angle, and perioperative or postoperative complications.

Functional outcomes at 12 months were categorized according to the percentage improvement in the WOMAC score. An improvement of ≥75% was considered excellent, 50-74% good, 25-49% fair, and <25% poor.

Data management and statistical analysis

Data entry and statistical analysis were performed using IBM SPSS Statistics for Windows, Version 25.0 (IBM Corp., Armonk, New York, United States). Continuous variables were summarized as mean±standard deviation (SD), whereas categorical variables were reported as frequencies and percentages.

The distribution of the data was evaluated using the Shapiro-Wilk test to assess normality. For continuous variables demonstrating normal distribution, including medial joint space width and tibiofemoral angle, preoperative and postoperative comparisons were conducted using paired t-tests. For variables that did not follow a normal distribution, such as VAS and WOMAC scores, the Wilcoxon signed-rank test was employed.

Associations between categorical variables, including sex, Kellgren-Lawrence grade, and functional outcome categories, were analyzed using the chi-squared test. Statistical significance was defined as a p-value less than 0.05.

Ethical considerations

Prior approval for the study was granted by the Research and Institutional Review Committee (IRC) of Nepal Medical College and Teaching Hospital (approval number: ThesisProp 41-076/077) before the research was initiated. All participants provided written informed consent prior to enrollment. Patient information was handled with strict confidentiality throughout the study period, and participants were clearly informed that they could withdraw from the study at any time without any impact on their ongoing medical care.

## Results

Baseline characteristics

A total of 42 patients diagnosed with medial compartment knee OA underwent PFO and successfully completed the 12-month follow-up period. The mean age of the participants was 56.90±9.33 years (95% CI: 54.00-59.81). Among them, 24 were male (57.1%), and 18 were female (42.9%). The mean BMI was 27.84±2.48 kg/m² (95% CI: 27.06-28.61). The right knee was involved in 23 patients (54.8%), while 19 patients (45.2%) had involvement of the left knee. A summary of the baseline demographic characteristics of the participants is presented in Table 1.

**Table 1:** Demographic characteristics of the study population (n=42) BMI: body mass index

Variable	Value
Age (years)	56.90±9.33
BMI (kg/m²)	27.84±2.48
Male	24 (57.1%)
Female	18 (42.9%)
Right knee	23 (54.8%)
Left knee	19 (45.2%)

**Figure 4:**
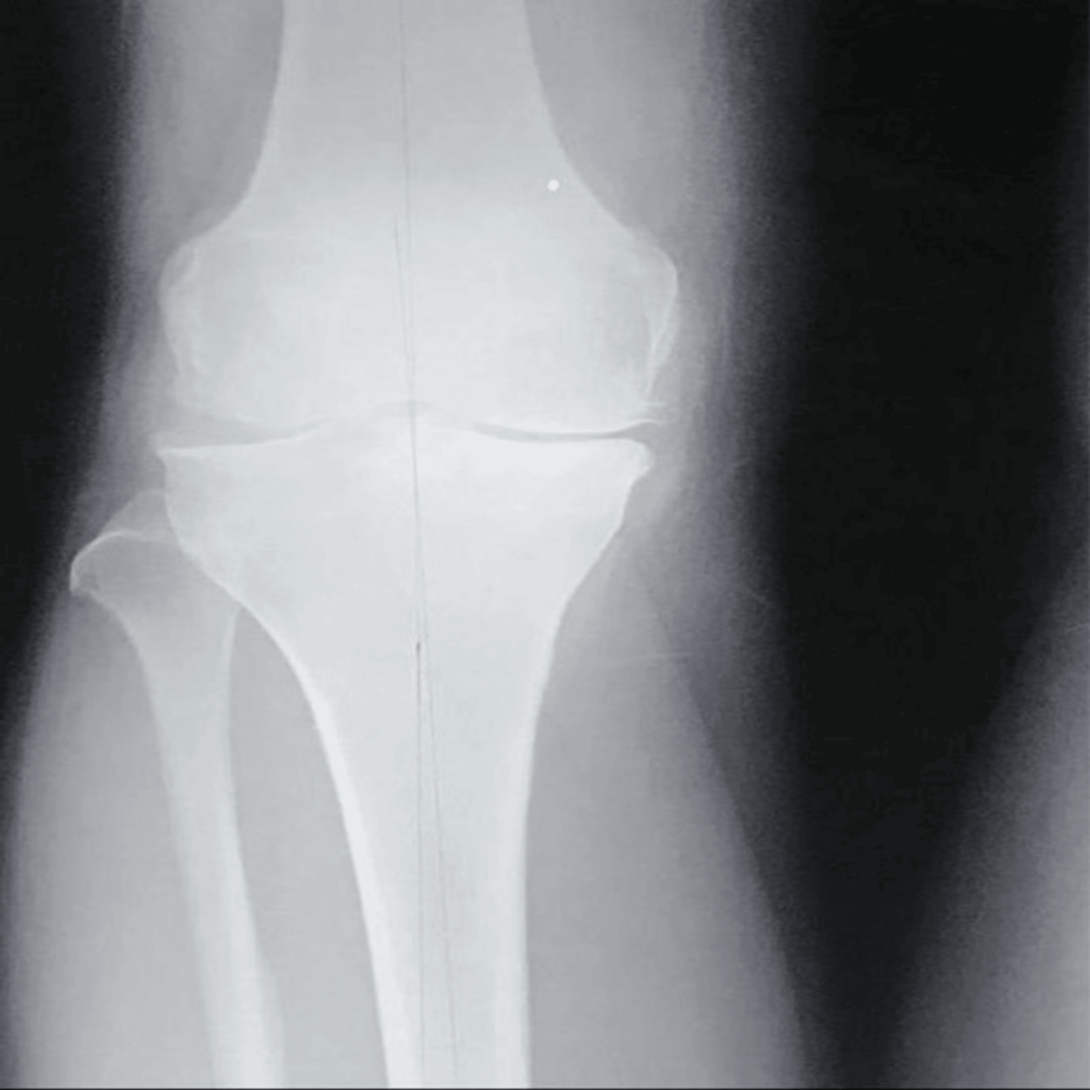
Preoperative anteroposterior radiograph demonstrating medial compartment osteoarthritis with medial joint space narrowing

**Figure 5:**
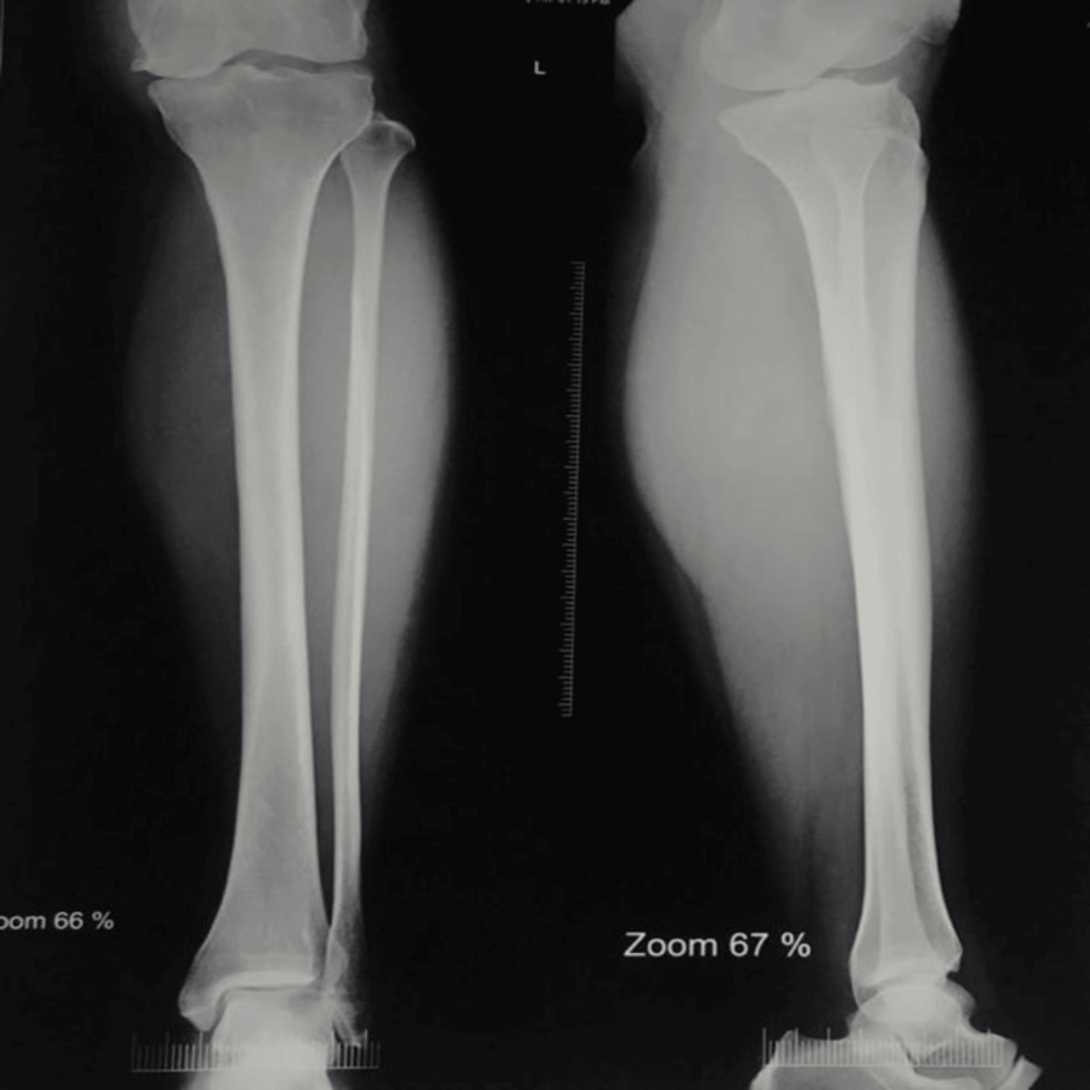
Preoperative full-length standing radiograph showing varus alignment and mechanical axis deviation toward the medial compartment

Preoperative radiographs demonstrated medial compartment joint space narrowing and varus deformity consistent with medial compartment OA (Figure 4). Full-length standing radiographs further confirmed mechanical axis deviation toward the medial compartment (Figure 5).

Preoperative clinical and radiological characteristics

The mean duration of symptoms was 4.80±2.04 years (95% CI: 4.16-5.43). The mean preoperative VAS score was 7.29±1.13 (95% CI: 6.93-7.64), and the mean WOMAC total score was 75.36±8.68 (95% CI: 72.65-78.06). Radiological assessment demonstrated a mean medial joint space width of 2.04±0.39 mm (95% CI: 1.92-2.16) and a mean tibiofemoral varus angle of 8.44°±2.29° (95% CI: 7.73-9.15). These baseline clinical and radiological characteristics are presented in Table 2.

**Table 2:** Preoperative clinical and radiological characteristics VAS: Visual Analog Scale; WOMAC: Western Ontario and McMaster Universities Osteoarthritis Index; SD: standard deviation

Variable	Mean±SD
Duration of symptoms (years)	4.80±2.04
VAS score	7.29±1.13
WOMAC total score	75.36±8.68
Medial joint space width (mm)	2.04±0.39
Tibiofemoral varus angle (°)	8.44±2.29

Kellgren-Lawrence grading

A total of 22 patients (52.4%) had Kellgren-Lawrence grade II OA, while 20 patients (47.6%) had grade III OA. The distribution of OA severity is shown in Table 3.

**Table 3:** Distribution of Kellgren-Lawrence grading

Grade	Frequency (%)
Grade II	22 (52.4%)
Grade III	20 (47.6%)

Pain assessment (VAS)

The Shapiro-Wilk testing indicated that VAS scores were not normally distributed (p<0.05); therefore, the Wilcoxon signed-rank test was used. A significant reduction in pain was observed at both follow-up time points (p<0.001). The mean VAS score decreased from 7.29±1.13 (95% CI: 6.93-7.64) preoperatively to 4.38±1.45 (95% CI: 3.93-4.83) at six months and 3.33±1.76 (95% CI: 2.78-3.88) at 12 months. Paired analysis demonstrated a mean reduction of 2.91 points at six months (95% CI: 2.62-3.19; p<0.001) and 3.95 points at 12 months (95% CI: 3.62-4.29; p<0.001). These results are presented in Table 4.

**Table 4:** Comparison of VAS score over time VAS: Visual Analog Scale; SD: standard deviation; IQR: interquartile range

Time point	Mean±SD	Median (IQR)	Test used	P-value
Preoperative	7.29±1.13	7.00 (6.00-8.00)	-	-
6 months	4.38±1.45	4.00 (3.00-5.00)	Wilcoxon signed-rank	<0.001
12 months	3.33±1.76	3.00 (2.00-5.00)	Wilcoxon signed-rank	<0.001

Functional outcome (WOMAC score)

WOMAC scores also demonstrated non-normal distribution; therefore, the Wilcoxon signed-rank test was used. Significant improvement in WOMAC scores was observed (p<0.001). The mean WOMAC score decreased from 75.36±8.68 (95% CI: 72.65-78.06) preoperatively to 43.81±11.72 (95% CI: 40.16-47.46) at six months and 33.60±12.94 (95% CI: 29.56-37.63) at 12 months. Paired analysis showed a mean reduction of 31.55 points at six months (95% CI: 29.42-33.68; p<0.001) and 41.76 points at 12 months (95% CI: 38.85-44.68; p<0.001). These findings are shown in Table 5.

**Table 5:** Comparison of WOMAC total score over time WOMAC: Western Ontario and McMaster Universities Osteoarthritis Index; SD: standard deviation; IQR: interquartile range

Time point	Mean±SD	Median (IQR)	Test used	P-value
Preoperative	75.36±8.68	75.00 (67.75-80.25)	-	-
6 months	43.81±11.72	42.50 (35.75-50.00)	Wilcoxon signed-rank	<0.001
12 months	33.60±12.94	33.50 (25.00-42.00)	Wilcoxon signed-rank	<0.001

Radiological outcomes

Radiological parameters including medial joint space width and tibiofemoral varus angle demonstrated normal distribution; therefore, paired t-tests were applied.

Medial joint space width

**Figure 6:**
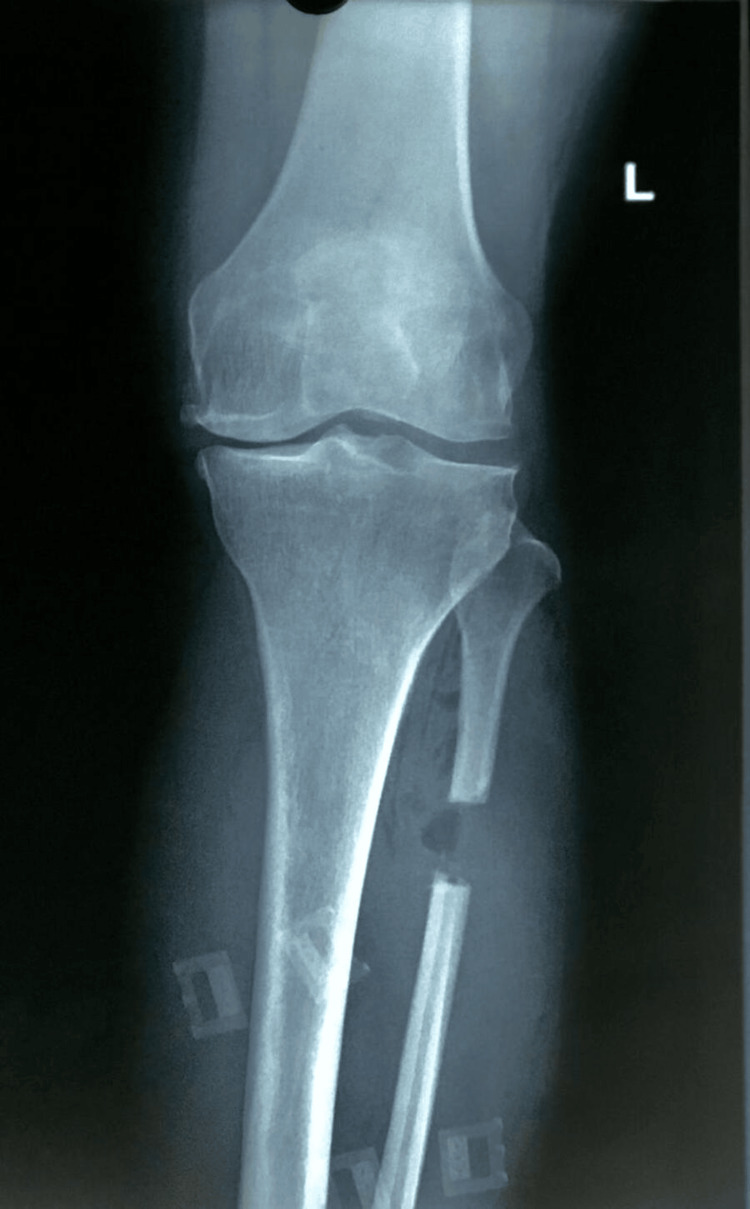
Postoperative radiograph demonstrating medial joint space widening

A significant postoperative increase in medial joint space width was observed (p<0.001). The mean value increased from 2.04±0.39 mm (95% CI: 1.92-2.16) preoperatively to 3.22±0.51 mm (95% CI: 3.06-3.38) at six months and 3.74±0.46 mm (95% CI: 3.59-3.88) at 12 months. Paired analysis showed a mean increase of 1.18 mm at six months (95% CI: 1.07-1.28; p<0.001) and 1.70 mm at 12 months (95% CI: 1.61-1.79; p<0.001). These findings are summarized in Table 6. A postoperative radiograph demonstrating medial joint space widening is illustrated in Figure 6.

**Table 6:** Change in medial joint space width SD: standard deviation; mm: millimeters

Time point	Mean±SD (mm)	P-value
Preoperative	2.04±0.39	-
6 months	3.22±0.51	<0.001
12 months	3.74±0.46	<0.001

Tibiofemoral varus angle

A significant correction in varus alignment was observed (p<0.001). The mean tibiofemoral angle decreased from 8.44°±2.29° (95% CI: 7.73-9.15) preoperatively to 5.38°±2.42° (95% CI: 4.63-6.14) at six months and 4.40°±2.43° (95% CI: 3.64-5.16) at 12 months. Paired analysis demonstrated a mean reduction of 3.06° at six months (95% CI: 2.76-3.35; p<0.001) and 4.04° at 12 months (95% CI: 3.68-4.40; p<0.001). These results are presented in Table 7.

**Table 7:** Change in tibiofemoral varus angle SD: standard deviation; °: degrees

Time point	Mean±SD (°)	P-value
Preoperative	8.44±2.29	-
6 months	5.38±2.42	<0.001
12 months	4.40±2.43	<0.001

Functional outcome categorization

At 12 months, 26 patients (61.9%) achieved excellent outcomes, 14 patients (33.3%) had good outcomes, and two patients (4.8%) showed fair outcomes. No patients had poor outcomes. The distribution of functional outcomes is presented in Table 8.

**Table 8:** Functional outcome category WOMAC: Western Ontario and McMaster Universities Osteoarthritis Index

Outcome	Frequency (%)
Excellent	26 (61.9%)
Good	14 (33.3%)
Fair	2 (4.8%)
Poor	0

Postoperative complications

The overall complication rate was 19% (8/42 patients). Most complications were minor and transient. Transient peroneal nerve palsy occurred in three patients (7.1%), and superficial wound infection occurred in one patient (2.4%). Persistent knee pain (n=4; 9.5%) was considered an unfavorable outcome rather than a complication. All cases of peroneal nerve palsy resolved spontaneously during follow-up. The complication profile is summarized in Table 9.

**Table 9:** Postoperative complications

Complication	Frequency (%)
None	34 (81%)
Superficial infection	1 (2.4%)
Transient peroneal nerve palsy	3 (7.1%)
Persistent pain	4 (9.5%)

Association analysis

No statistically significant association was observed between sex and functional outcome (χ²=5.385; p=0.068) or between Kellgren-Lawrence grade and functional outcome (p>0.05).

Operative characteristics

The mean operative time was 36.43±7.12 minutes (95% CI: 34.21-38.65), the mean hospital stay was 2.95±1.18 days (95% CI: 2.58-3.32), and the mean intraoperative blood loss was 47.88±16.32 mL (95% CI: 42.80-52.97). These operative details are summarized in Table 10.

**Table 10:** Operative characteristics SD: standard deviation; mL: milliliters

Variable	Mean±SD
Operative time (minutes)	36.43±7.12
Hospital stay (days)	2.95±1.18
Blood loss (mL)	47.88±16.32

## Discussion

This prospective single-arm interventional study demonstrated that PFO was associated with significant improvements in pain, functional outcome, medial joint space width, and correction of varus deformity at six and 12 months of follow-up. These findings are consistent with the growing body of literature from Nepal, South Asia, and global cohorts supporting the role of PFO as a joint-preserving surgical option for medial compartment knee OA.

Pain and functional improvement

In this study, a significant reduction in pain was observed, with the mean VAS score decreasing from 7.29±1.13 before surgery to 3.33±1.76 at the 12-month follow-up (p<0.001). Similarly, the mean WOMAC score improved from 75.36±8.68 to 33.60±12.94 (p<0.001). These results closely mirror findings from Joshi et al. in Nepal, who reported a reduction in VAS from 8.80±1.17 to 2.60±0.96 following PFO [9]. Similarly, Bajaj et al. demonstrated significant postoperative improvement in VAS and Modified Oxford Knee Scores at 12 months [10], while Dhamsania et al. from India observed VAS reduction from approximately 6.3 to 2.8 and substantial improvement in Knee Society Scores (KSS) after PFO [11]. A large Indian prospective cohort by Kumar et al. also showed a significant reduction in VAS and marked improvement in KSS and functional scores at 12 months [12].

Systematic reviews and meta-analyses further reinforce these findings. Ashraf et al. reported pooled significant reductions in VAS and improvements in KSS across 12 clinical studies [7]. Sugianto et al. demonstrated a pooled mean VAS reduction of approximately −4.13 points with meaningful functional gains [13], and Jiang et al. reported similarly large pooled reductions in pain and improvements in knee function across multiple studies [14]. These findings suggest that the magnitude of pain relief and functional recovery observed in our cohort is consistent with both regional and international evidence.

However, not all studies have reported uniformly positive results. Sabir et al. found no statistically significant improvement in VAS or KSS at 18 months of follow-up in their Indian cohort [15]. This discrepancy highlights the importance of appropriate patient selection and suggests that variability in baseline alignment, severity, and surgical technique may influence outcomes.

Radiological outcomes and biomechanical implications

Our study demonstrated significant widening of the medial joint space (2.04 mm to 3.74 mm) and reduction in tibiofemoral varus angle (8.44° to 4.40°) at 12 months (p<0.001). These radiological findings are comparable to those reported by Joshi et al., who observed medial joint space widening from 2.14 mm to 4.13 mm [9]. Dhamsania et al. similarly reported increased medial joint space and improved medial-to-lateral joint space ratio following PFO [11].

The mechanical rationale of PFO, namely, lateral column decompression leading to the redistribution of load, has been consistently supported in the literature. Vaish et al. described the fibula as a lateral strut whose resection reduces lateral constraint, shifts load away from the overloaded medial compartment, and contributes to varus correction and pain relief [16]. Singh also reported the opening of the medial joint space and improved weight-bearing axis in their mid-term study [17].

Meta-analytic evidence corroborates these radiological improvements. Sugianto et al. reported significant improvement in medial-to-lateral joint space ratio and alignment parameters [13], while Jiang et al. demonstrated a pooled medial joint space increase of approximately +2.66 mm along with improvements in mechanical alignment [14]. The PLOS One study by Liu et al. further emphasized that preoperative radiographic factors such as the hip-knee-ankle (HKA) angle and medial joint space width significantly influenced postoperative outcomes, reinforcing the biomechanical basis of PFO [18].

Interestingly, comparative evidence suggests that PFO may achieve radiological and functional improvements comparable to unicompartmental knee arthroplasty (UKA). Dai et al. found no significant difference between PFO and UKA in VAS, KSS, range of motion, or alignment measures, while PFO demonstrated shorter operative time and lower blood loss [19]. These findings align with the minimally invasive profile observed in our study.

Complications and safety profile

The overall complication rate in our study was 19%, predominantly minor and transient. Transient peroneal nerve palsy occurred in 7.1% of patients, with no permanent deficits. This is consistent with pooled complication rates reported by Sugianto et al., where peroneal nerve paresthesia (~5.9%) and palsy (~2.3%) were among the most common but typically transient complications [13]. Jiang et al. also reported low overall complication rates across pooled studies [14].

In contrast, Adhikari et al. reported a relatively higher rate of transient peroneal neuropraxia (~29.6%) in patients undergoing combined arthroscopic debridement and PFO, although all cases resolved spontaneously [20]. Sabir et al. also reported neurological symptoms in a subset of patients [15]. These findings emphasize the importance of meticulous surgical technique and appropriate osteotomy level to minimize nerve-related complications.

Overall interpretation and clinical relevance

Taken together, the present findings align with most Nepalese, South Asian, and global studies demonstrating significant pain relief, functional improvement, medial joint space widening, and alignment correction following PFO [7,9-14]. The procedure's short operative time, minimal blood loss, and limited hospital stay observed in our cohort further support evidence from comparative analyses highlighting its potentially lower-cost nature and lower surgical burden relative to arthroplasty [19].

PFO may be particularly useful in patients who are not suitable candidates for or cannot afford arthroplasty, especially in resource-limited healthcare settings.

While some contradictory evidence exists [15], the preponderance of available data, including systematic reviews and meta-analyses, suggests that PFO is associated with meaningful short- to mid-term benefits in selected patients with medial compartment OA. However, as emphasized by Ashraf et al. and other reviewers, the overall quality of evidence remains low to moderate due to the predominance of observational studies and limited long-term data [7].

Given the single-arm design of the present study, the observed improvements should be interpreted as associations rather than definitive causal effects.

Although the results of this study indicate that PFO may represent a safe and useful surgical option, particularly in resource-constrained healthcare settings such as Nepal, additional evidence from larger multicenter randomized studies with longer follow-up is required to evaluate the long-term sustainability of correction, patterns of disease progression, and appropriate criteria for patient selection.

Limitations

Although the findings are encouraging, several limitations should be considered when interpreting the results. The study was conducted at a single center with a relatively small number of participants and employed a single-arm design without a control group, limiting comparison with other surgical procedures such as HTO. In addition, the follow-up period was limited to 12 months, which restricts the evaluation of long-term outcomes and progression of OA.

Furthermore, the absence of blinded outcome assessment may introduce observer bias, and the use of patient-reported outcome measures may be subject to response bias. Some degree of measurement bias in the radiological assessments cannot be entirely ruled out.

Future research should include larger study populations, extended follow-up durations, and comparative or randomized study designs, such as evaluating PFO against HTO, to better define appropriate patient selection and long-term clinical outcomes.

## Conclusions

PFO was associated with significant pain reduction, functional improvement, radiological correction of varus deformity, and acceptable complication rates at 12 months. The procedure appears to be a safe, minimally invasive, and potentially lower-cost surgical option for patients with medial compartment OA of the knee, particularly in resource-constrained settings. However, these findings should be interpreted with caution due to the single-arm study design and lack of a comparator group. These findings support the role of PFO as a joint-preserving treatment option and provide valuable local evidence to guide orthopaedic practice in Nepal.
